# Robust production of uniform human cerebral organoids from pluripotent stem cells

**DOI:** 10.26508/lsa.202000707

**Published:** 2020-04-17

**Authors:** Adam A Sivitilli, Jessica T Gosio, Bibaswan Ghoshal, Alesya Evstratova, Daniel Trcka, Parisa Ghiasi, J Javier Hernandez, Jean Martin Beaulieu, Jeffrey L Wrana, Liliana Attisano

**Affiliations:** 1 Department of Biochemistry, University of Toronto, Toronto, Canada; 2 Donnelly Centre, University of Toronto, Toronto, Canada; 3 Department of Molecular Genetics, University of Toronto, Toronto, Canada; 4 Center for Systems Biology, Lunenfeld-Tanenbaum Research Institute, Mount Sinai Hospital, Toronto, Canada; 5 Department of Pharmacology and Toxicology, University of Toronto, Toronto, Canada

## Abstract

Human cerebral organoid (hCO) models offer the opportunity to understand fundamental processes underlying human-specific cortical development and pathophysiology in an experimentally tractable system. Although diverse methods to generate brain organoids have been developed, a major challenge has been the production of organoids with reproducible cell type heterogeneity and macroscopic morphology. Here, we have directly addressed this problem by establishing a robust production pipeline to generate morphologically consistent hCOs and achieve a success rate of >80%. These hCOs include both a radial glial stem cell compartment and electrophysiologically competent mature neurons. Moreover, we show using immunofluorescence microscopy and single-cell profiling that individual organoids display reproducible cell type compositions that are conserved upon extended culture. We expect that application of this method will provide new insights into brain development and disease processes.

## Abstract (precis)

By leveraging advances in 3D stem cell culture techniques, the authors describe, characterize and validate a novel platform to efficiently generate morphologically consistent human forebrain cerebral organoids.

## Introduction

The development, patterning, and homeostatic maintenance of the human brain is complex and while considerable insights into mechanisms driving these processes have been obtained from studies in model organisms, species-specific differences in brain development and function can make it challenging to apply results from animal models to humans. Accordingly, understanding the molecular basis underlying normal development, disease progression, and therapeutic options for human brain-associated diseases, including cancer, requires human models.

The ability to generate brain organoids derived from human pluripotent stem cells provides an unprecedented opportunity to study context-dependent human disease pathologies in an experimentally tractable system. Indeed, this approach has provided insights into alterations associated with Alzheimer’s, blindness, autism spectrum disorder, Zika virus infection, and others (Lancaster & Knoblich, 2014b; Quadrato et al, 2016; Di Lullo & Kriegstein, 2017; Amin & Pasca, 2018; Rossi et al, 2018; Chen et al, 2019). A variety of protocols to generate brain organoids have been developed, but the considerable variability and heterogeneity between individual organoids obtained using these methods limits the utility of the model for studying disease mechanisms or for examining the therapeutic potential of new drug candidates. Here, we establish a robust protocol to efficiently and reproducibly generate mature, consistent (i.e., uniform) human cerebral organoids (hCOs). By optimizing an established protocol for self-patterned whole-brain organoids (Lancaster et al, 2013; Lancaster & Knoblich, 2014a), we generated phenotypically consistent forebrain organoids with reproducible morphologies and cell-type compositions. Thus, this protocol is ideally suited for studying mechanisms underlying human diseases and for investigation of potential novel therapeutic options in an experimentally tractable system.

## Results

### Optimization of cerebral organoid production

**Figure 1.**
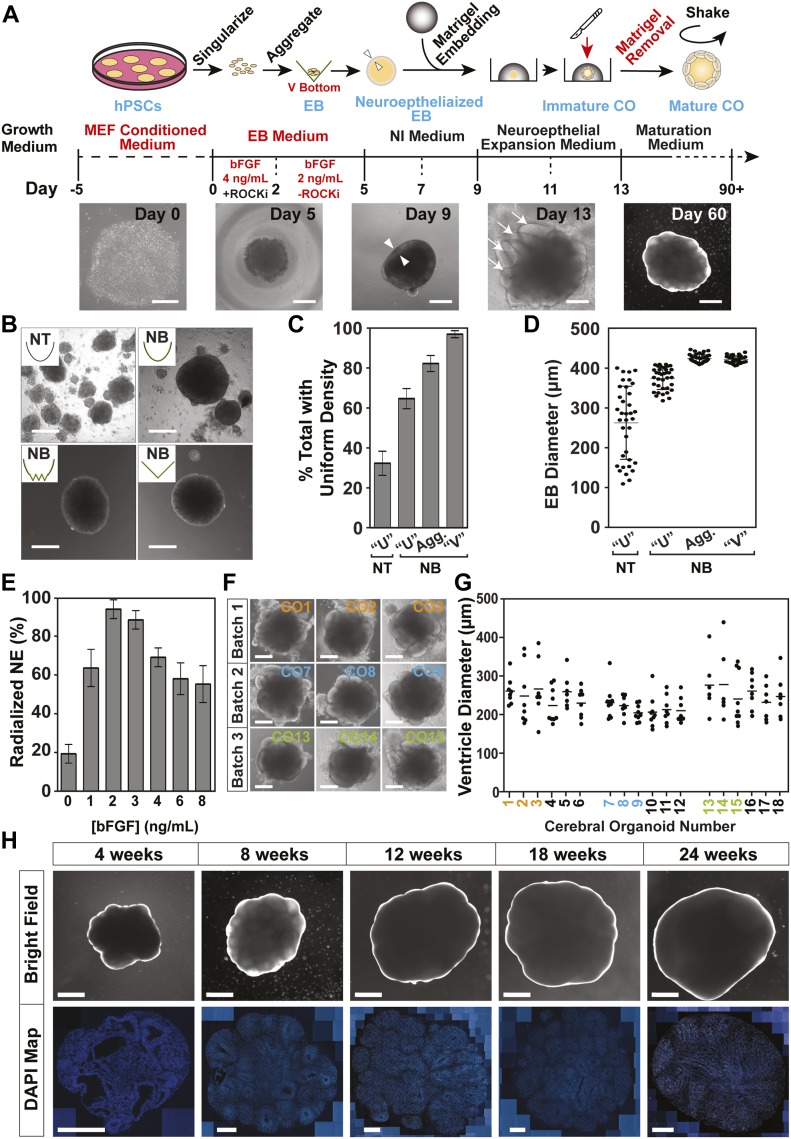
Generation of hCOs from H9 ESCs. **(A)** A schematic depicting the main steps for human cerebral organoid (hCO) production. Representative bright-field images of morphological changes are shown below. Triangles (Day 9) mark the inner and outer edge of the neuroepithelial ring, and arrows (Day 13) indicate early ventricle structures. Scale bars: 250 μm for days 0, 5, 9, and 13 and 1 mm for Day 60. **(B, C, D)** The effect of well shape and surface coating on embryoid body (EB) formation was assessed on Day 5. **(B)** Representative bright-field images of EBs generated using the indicated plate format. Scale bar = 250 μm. Non-treated (NT), nonbinding (NB). **(C)** Percent of cell aggregates displaying uniform density as assessed using phase-contrast microscopy is plotted as the mean ± SD (n = 3). **(D)** Individual EB diameters (black circles) and the mean (horizontal dash) ± SD (n ≥ 30/condition) is plotted. **(E)** Percent of total EBs displaying radialization neuroepithelium on Day 5 at the indicated bFGF concentrations are plotted as mean ± SD (n = 3). **(F, G)** Analysis of ventricle formation on Day 13. **(F)** Representative bright-field images of COs before Matrigel extraction from three independent batches are shown. Scale bar = 500 μm. **(G)** Quantification of the diameter of individual ventricle-like structures (black circles) from three independent batches is plotted with the mean diameter marked (horizontal dash). **(F)** Colored numbering corresponds to images in panel (F). **(H)** Macroscopic organization in H9-derived hCOs. Representative bright-field (Scale bar = 1 mm) images of hCOs in suspension culture at 4, 8, 12, 18, and 24 wk of culture (top) with corresponding sections stained with DAPI to mark nuclei (bottom). Scale bar = 500 μm.

To establish a method to reproducibly generate uniform brain organoids (Fig 1A), we explored modifications to a previously established protocol for generating self-patterned whole-brain organoids (Lancaster et al, 2013; Lancaster & Knoblich, 2014a), which yields organoids with variable morphology and cell type composition (Quadrato et al, 2017; Velasco et al, 2019; Yoon et al, 2019). We primarily used female H9 human embryonic stem cells (hESCs) and validated results in a male hESC model (H1; see below). To begin, we first optimized embryoid body (EB) generation by plating singularized H9 cells into 96-well plates with variable geometries and surface coatings and quantitatively examined cell aggregates after 5 d. In contrast to the irregular clusters observed in traditional U-bottom dishes with non-treated (unmodified polystyrene) or nonbinding (Ultra Low Attachment) surface coatings, EB aggregates that were formed in nonbinding plates with V-bottom or Aggrewell 800 (comprising multiple V-shaped indentations) geometries, formed similarly sized spheres of 400–450-μm diameter in each V-shaped indentation, all of which displayed similar opacity under bright-field microscopy (Fig 1B–D). Although we were able to obtain consistent EB size using both the V-bottom and Aggrewell platforms, the Aggrewell system generated multiple EBs per well which when transferred for neuralization, resulted in further aggregation of multiple EBs. For this reason, we focused on the V-bottom nonbinding format for all subsequent studies as this streamlined selection of individual EBs.

**Figure S1.**
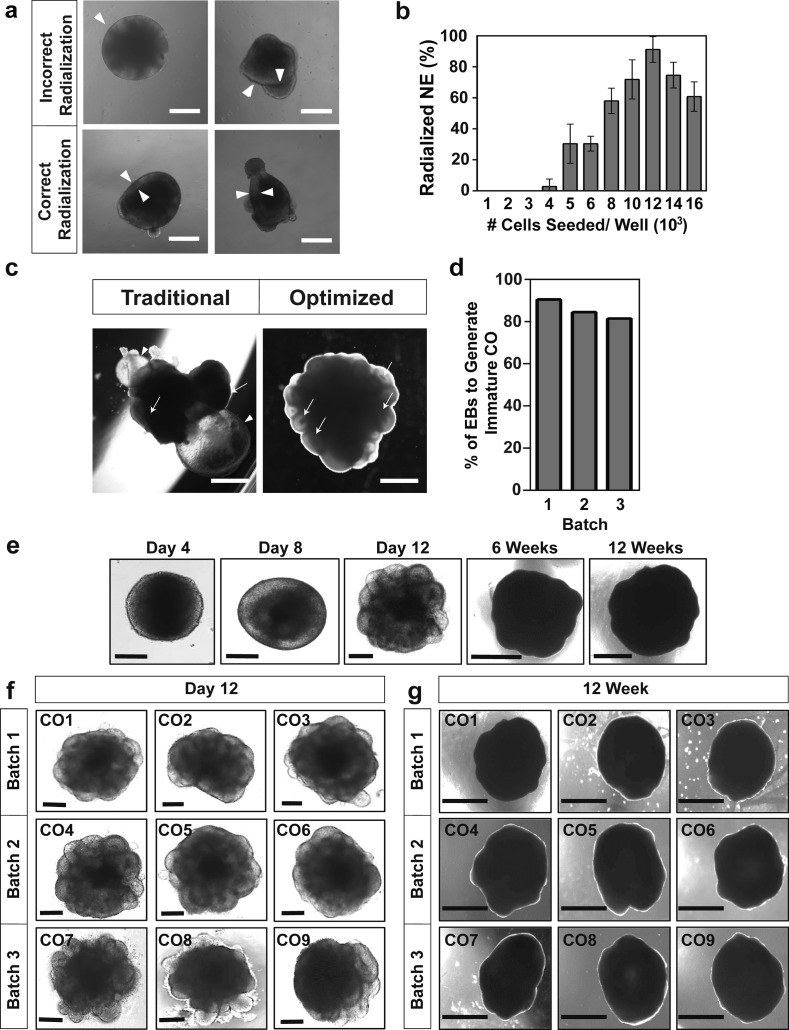
Morphological characterization of H9- and H1-derived human cerebral organoids (hCOs). **(A)** Representative bright-field images of failed (top panels) or successful (bottom panels) neuroepithelialization on Day 4 of neural induction from H9 ESCs. White triangles denote the neuroepithelial ring. Scale bar = 250 μm. **(B)** Percent of total EBs displaying radialization neuroepithelium on Day 5 at the indicated cell seeding concentrations is plotted as mean ± SD (n = 3). **(C)** Representative images of hCOs generated using the optimized and traditional CO differentiation pipelines. Arrows show ventricle structures and the arrowhead highlights a fluid-filled cyst structure on the organoid exterior. Scale bar = 1 mm. **(D)** Efficiency of H9-derived COs displaying ventricle ring structures on Day 13, before Matrigel extraction, from three independent batches of COs. **(E, F, G)** Generation of H1-derived hCOs. **(E)** Representative bright-field images of morphological changes at the indicated ages are shown. **(F, G)** Representative bright-field images of COs before Matrigel extraction on Day 12 (F) and at 12 wk (G) from three independent batches are shown. Scale bars: 200 μm for days 4–12 and 1 mm for 8 and 12 wk.

Basic FGF (bFGF) in the presence of Nodal/Activin SMAD signalling is important for maintenance of pluripotency in hESC cells (Vallier et al, 2005), whereas in the absence of SMAD signalling, FGF drives neuroectoderm induction (LaVaute et al, 2009). However, hESC aggregates display intrinsic suppression of BMP-SMAD signalling, so the addition of FGF is sufficient to drive neural induction (Coucouvanis & Martin, 1999; Khoo et al, 2005; LaVaute et al, 2009). Furthermore, although ROCK inhibitor (Y27) promotes survival and pluripotency of singularized hESC, it is not required for EB formation (Pettinato et al, 2014). Therefore, we removed Y27 after EBs formed (2 d) and titrated FGF doses for the following 3 d to determine the optimal concentration that supports the subsequent formation of radialized neuroepithelium after neural induction. We observed that after 3 d at 2 ng/ml, >90% of EBs displayed correctly radialized neuroepithelium (i.e., a uniform, smooth clearing at the periphery: Fig S1A), which decreased to 60% at bFGF concentrations of >6 ng/ml. In contrast, removal of bFGF strongly suppressed radialization to 20% (Fig 1E), consistent with the important role of FGF signalling in neurectoderm induction observed in vivo, and in hESC aggregates (Vallier et al, 2005; LaVaute et al, 2009). hCOs develop as self-organizing systems, and EB size has a significant impact on differentiation trajectories, with smaller EBs favouring ectoderm (Bauwens et al, 2008; Jang & Kim, 2010). Therefore, we also examined the effect of cell seeding density (1,000–16,000 cells/EB) on neural induction efficiency and observed peak efficiency at 12,000 cells/well (Fig S1B). Finally, we noted that when early hCOs were extracted from Matrigel and transferred to free-floating spinning cultures, excessive Matrigel impeded the growth of morphologically uniformly shaped hCOs. This prompted us to ensure complete removal of all Matrigel from hCOs before transfer to spinning culture. For this reason, we transitioned from using the “dimpled parafilm” method (Lancaster & Knoblich, 2014a) to embedding neuralized EBs in a Matrigel droplet immobilized on the surface of a four-well plate to facilitate complete extraction. Using all of the above optimized parameters, we assessed the morphology of 18 organoids, six from each of three separate batches on Day 13, just before transfer to spinning culture. These early organoids displayed 6–10 outer ventricle-like ring structures with an average diameter of approximately 250 μm in all batches (Fig 1F and G). We also noted a marked absence of the fluid-filled cyst structures (Fig S1C) commonly seen with other protocols (Lancaster et al, 2013; Lancaster & Knoblich, 2014a). Thus, by optimizing early EB formation and the neural induction phases of organoid production, we reproducibly generated morphologically uniform hCOs with a cumulative efficiency of 80–90%, while restricting unwanted non-neural differentiation (Fig S1D). Similar results were also obtained using H1 ESCs (Fig S1E–G).

### Characterization of cerebral organoid development and maturation

**Figure S2.**
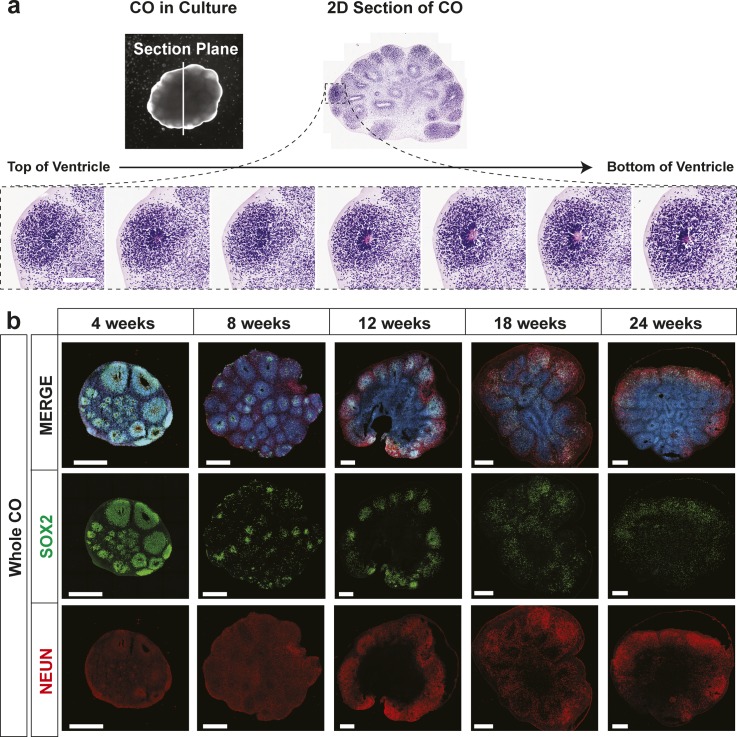
Human cerebral organoids (hCOs) mimic early human cortical development. **(A)** Orientation of ventricle unit in hCOs in 3D space. Visualization of serial sections of an hCO and a top view of a sectioned ventricle when mounted on a glass coverslip (top). H&E staining of seven serial sections of a ventricle to reconstruct the 3D structure (bottom). Scale bar = 250 μm. **(B)** Images of individual channels of the merged whole CO images shown in Fig 2. Localization of SOX2 (radial glia) and NeuN (neurons) in hCOs at 4, 8, 12, 18, and 24 wk of age, co-stained with DAPI, was visualized by immunofluorescence microscopy. Scale bar = 500 μm for whole COs.

**Figure 2.**
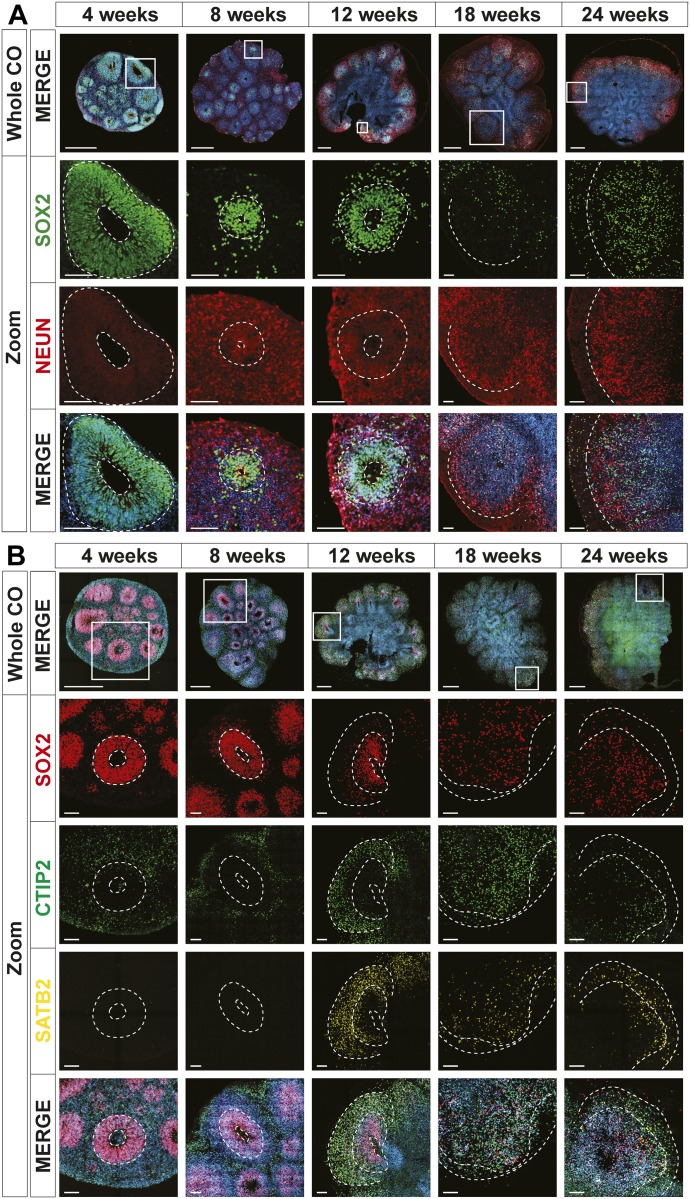
Human cerebral organoids (hCOs) mimic early human cortical development. The localization of SOX2 (radial glia), NeuN (neurons), CTIP2, and SATB2 (cortical layer markers) in hCOs at 4, 8, 12, 18, and 24 wk of age, co-stained with DAPI, was visualized by immunofluorescence microscopy. Images of whole hCOs (top) and magnified images (Zoom) of selected ventricles or regions (white box) at each time point are shown. White dashed lines mark ventricles (center ring) and the outer perimeter SOX+ radial glial cells (outer ring). White dashed lines mark the ventricle-like cavities (inner ring) and the outer perimeter of the SOX2+ layer in 4–12-wk COs or the SOX+ and outer edge of the cortical plate in 18- and 24-wk COs. **(A)** Ventricle-like structures are lost in older (18 and 24 wk) COs. **(B)** Expression of CTIP2 (gene name, *BCL11B*; deep layer cortical neuron marker) precedes that of SATB2 (upper layer cortical neuron marker), both of which are superficial to the SOX2+ ventricular zone in 4–12-wk COs, recapitulating in vivo cortical development, whereas in older COs (18 and 24 wk), this distinct separation is less evident. Scale bar = 500 μm for whole COs and 100 μm for magnified ventricles.

**Figure S3.**
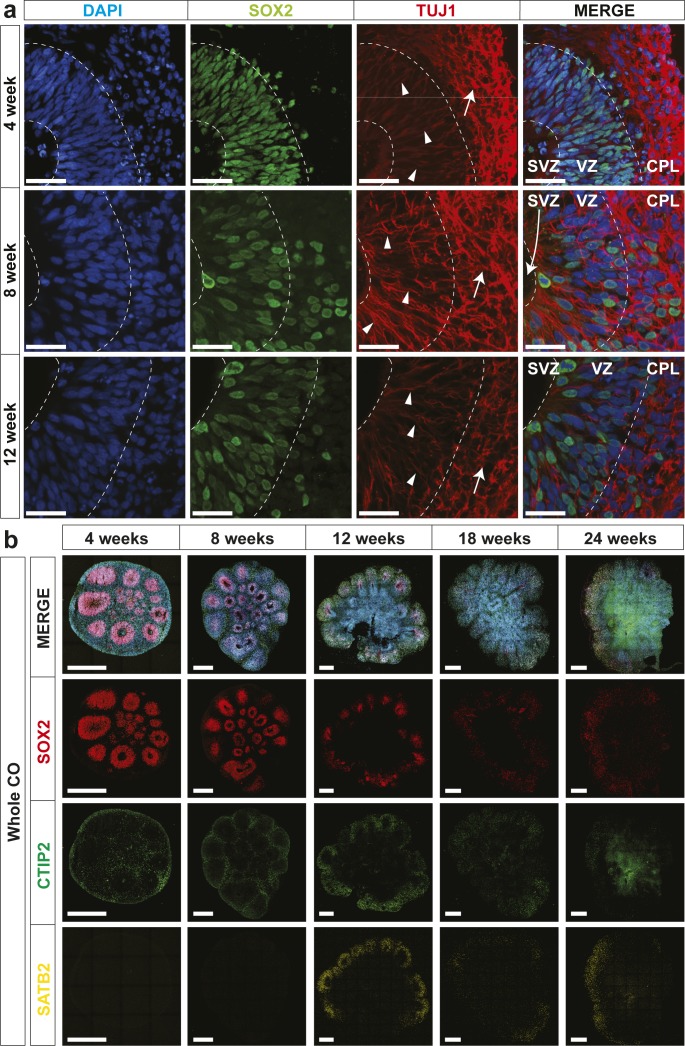
Characterization of ventricle-like structures in human cerebral organoids (hCOs). **(A)** Localization of SOX2 (radial glia) and TUJ1 (neurons) in hCOs at 4, 8, and 12 wk of age, co-stained with DAPI, was visualized by immunofluorescence microscopy. SOX2+ radial glial cells line the apical space of the ventricular zone adjacent to the hollow ventricle structure. Triangles denote TUJ1+ radial glial processes oriented perpendicular in the ventricular zone (VZ) and arrows denote TUJ1+ neurons with parallel processes in the cortical plate (CPL). The dashed line denotes layering present in hCOs. Scale bar = 50 μm. **(B)** Localization of SOX2 (radial glia), CTIP2, and SATB2 (cortical layer markers) in hCOs at 4, 8, 12, 18, and 24 wk of age, co-stained with DAPI, was visualized by immunofluorescence microscopy. Scale bar = 500 μm for whole COs. CP, cortical plate; SVZ, sub-ventricular zone; VZ, ventricular zone.

On Day 14, early organoids coated in Matrigel had a diameter of approximately 500 μm, and after careful excision from the Matrigel, they were transferred to spinning culture where they were left to mature as free-floating structures. By 12 wk, hCO diameters increased to roughly 3 mm after which little additional growth was observed for up to 24 wk of culture (Fig 1H). With the exception of an occasional loss due to the fusion of two organoids, virtually all hCOs continued to maturity. To assess morphogenesis, we next evaluated cell type–specific marker gene expression by immunofluorescence microscopy. Distinct ventricle-like structures (ventricular units) were abundant in early-stage hCOs (4–12 wk) and diminished in later stages (18 and 24 wk). Serial sectioning of ventricular units showed them to be spherical with a hollow center reminiscent of the apical space in ventricles in the developing cortex (Fig S2A). Staining with SOX2, which marks radial glial (RG) cells, showed expression in cells lining the ventricular space similar to in vivo (Figs 2A and S2B), with most ventricular units displaying robust SOX2+ staining at 4 wk. However, by 12 wk, SOX2+ ventricular units were more variable and in older hCOs were localized to the outer regions. In contrast, NeuN, which marks mature neurons, was low in 4-wk-old hCOs but was readily detected in hCOs 8 wk or older, where it was present outside of the SOX2 expression domain (Figs 2A and S2B). Similar to the in vivo orientation, TUJ1, a neurofilament protein, marked RG processes running perpendicular to the ventricular zone as early as 4 wk (Fig S3A). Outside of the RG cells, parallel TUJ1+ processes were observed in superficially localized neurons. The cortical layer markers, CTIP2 (gene name, *BCL11B*) and SATB2, which identify layers V (deep) and II–IV (upper), respectively, were also detected superficial to SOX2 staining in early hCOs, with CTIP2 detected as early as 4 wk, and SATB2 first evident at 12 wk (Figs 2B and S3B). This temporal sequence is consistent with the timing of in vivo development in which formation of deep layers precedes that of upper layer neurons. These studies further revealed that in older hCOs, although markers of RG cells and mature neurons were maintained, the cells expressing the markers became intermingled, and the organized layering of ventricular units evident in earlier hCOs was lost, as reported previously (Watanabe et al, 2017). Altogether, this analysis demonstrates that the hCOs generated using the optimized pipeline display organization and patterning reminiscent of that observed in vivo (Molnar et al, 2019).

### Single-cell profiling of hCOs

**Figure 3.**
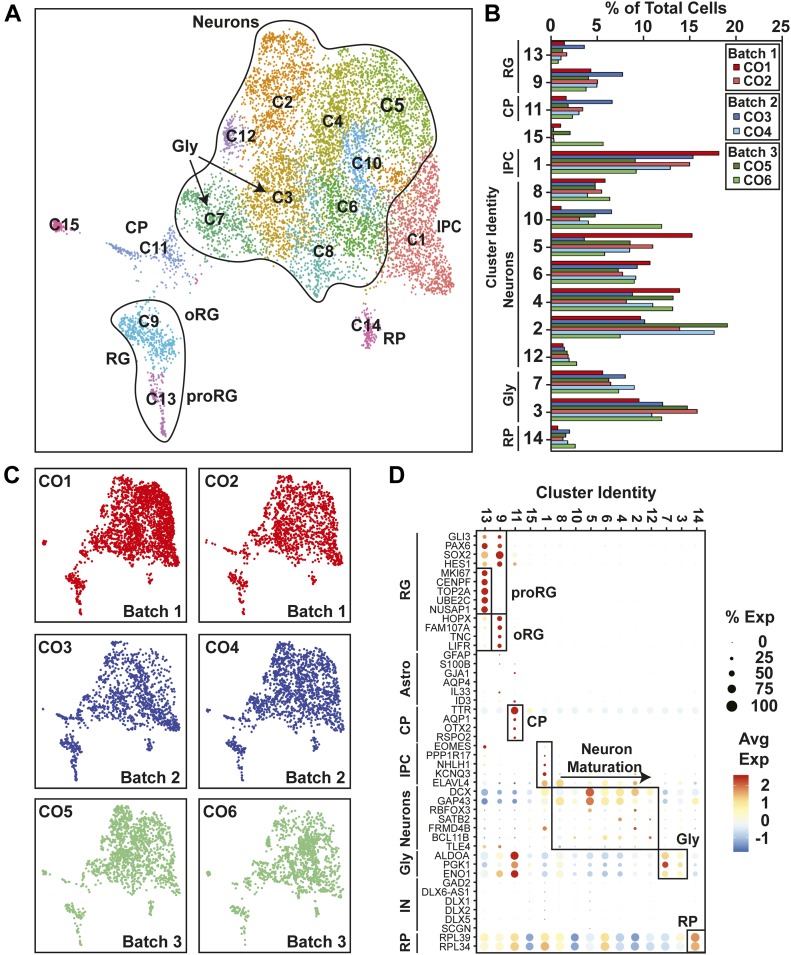
Uniform cell type composition in 12 human cerebral organoids (hCOs) is revealed by single-cell RNA sequencing. **(A)** A Uniform Manifold Approximation and Projection plot from unsupervised clustering of single-cell RNA sequencing data from six 12-wk-old hCOs obtained from three separate batches (CO1-CO6) (10,985 cells) is shown. Cluster identities are indicated. **(B)** Cluster frequency analysis depicting the percentage of cells in each individual organoid that contributed to each cluster. **(A, C)** Individual 12-wk-old hCOs (CO1-CO6) are plotted in the Uniform Manifold Approximation and Projection axis defined in panel (A). **(A, D)** A dot blot indicates the expression of cell type–specific marker genes for all clusters in panel (A).

**Figure S4.**
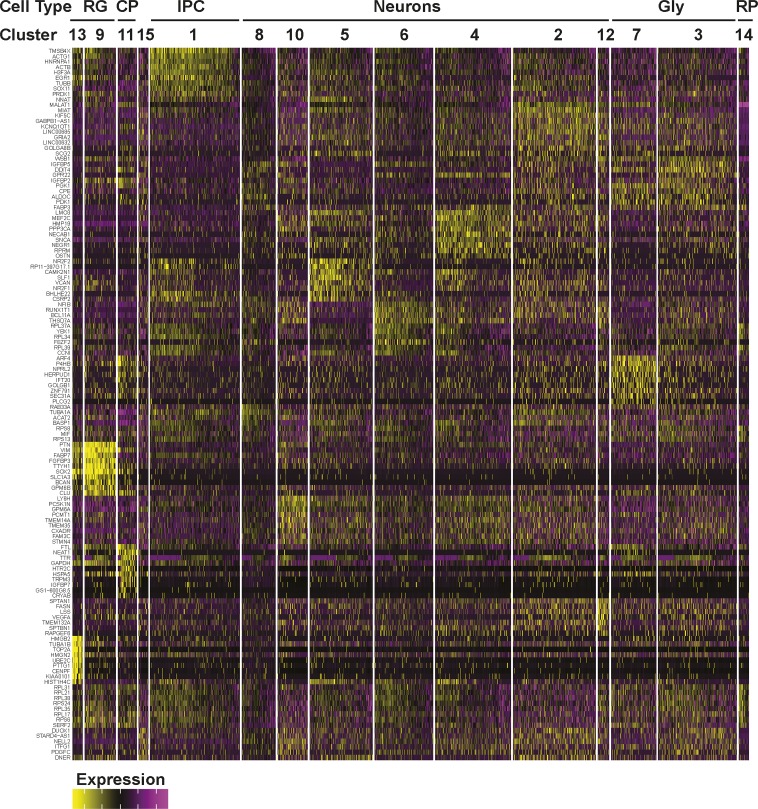
Gene expression heat map from 12-wk-old COs. The top 10 most differentially expressed genes per cluster are shown.

To gain a comprehensive view of cell types present in individual hCOs, we performed single-cell RNA sequencing (scRNA-seq) on individual organoids at 12, 18, and 24 wk of culture. Unsupervised clustering was performed on the gene expression profiles and data were visualized using Uniform Manifold Approximation and Projection (UMAP) plots, with single-cell metrics and sequencing data quality summarized in Table S1. Cell types were then identified by comparing the differentially expressed genes in each of the clusters to known cell type–specific marker genes. Profiling of six organoids derived from three separate batches at 12 wk of age identified 15 distinct clusters (Figs 3A–C and S4). This included RG cells (*SOX2*, *PAX6*, *HES1*, and *GLI3*) of both proliferative (proRG: cluster 13, *MKI67*, *CENPF*, and *TOP2A*) and outer RG (oRG; cluster 9, *HOPX*, *FAM107A*, *TNC*, and *LIFR*) subtypes (Pollen et al, 2015), with some overlap between these classes, consistent with RG cell function. RG cells give rise to neurons by transitioning through a transcriptionally distinct intermediate progenitor cell state marked by the expression of *EOMES* and *PPP1R17* (Pollen et al, 2015), which mapped to cluster 1. Neurons comprised the majority (74%) of the cells, encompassing clusters 2–8, 10, and 12. These included immature neurons expressing high levels of *DCX* and *GAP43*, as well as more mature neurons that expressed deep and superficial layer markers such as *SATB2* and *BCL11B* (CTIP2). Two of the neuronal clusters (3 and 7) displayed a particularly high level of glycolytic (Gly) marker genes (*ALDOA*, *PGK1*, and *ENO1*), a neuronal subset also recently reported in hCOs (Pollen et al, 2019). Interestingly, cells expressing markers of mature astrocytes (*GFAP* and *S100B*) were rare (Fig 3D), suggesting neurogenesis is the primary process occurring in the first 12 wk of culture. In addition to RG cells and neurons, we identified cluster 11 as choroid plexus (CP), with cells expressing characteristic CP markers (*TTR*, *AQP1*, *OTX2*, and *RSPO2*), whereas in one small cluster (#14), the differentially expressed genes were almost exclusively ribosomal proteins. Of note, the hCOs comprised predominantly dorsal forebrain neurons as markers of midbrain and hindbrain (*HOXA3*, *HOXB3*, *IRX2*, *EN2*, *PAX2*, and *GBX2*) were not detected. Moreover, there was a complete absence of markers identifying mesenchymal lineages (*MYOG*, *MYH1*, *DCN*, and *BGN*) or retinal cells (*OPN1SW*, *RCVRN*, *TULP1*, and *ROM1*) indicating the fidelity of our optimized pipeline in promoting forebrain-specific differentiation.

Table S1 Single-cell RNA sequencing data analyses and statistics.

**Figure S5.**
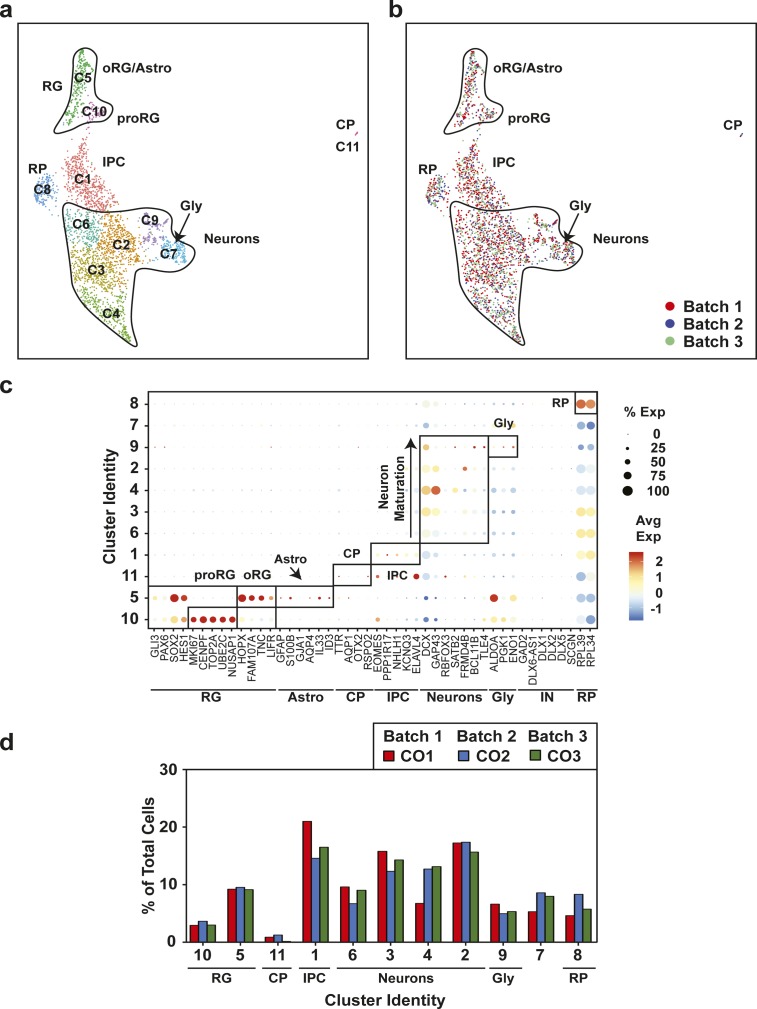
Uniform cell type composition in 18-wk-old human cerebral organoids (hCOs) is revealed by single-cell RNA sequencing. **(A)** Uniform Manifold Approximation and Projection plots from unsupervised clustering of single-cell RNA sequencing data from three hCOs from three separate batches (CO1-CO3) (12,000 cells) are shown. Cluster identities are indicated. Emergence of an astrocyte population is observed in the oRG/astro cluster 10. **(B)** A dot blot indicates the expression of cell type–specific marker genes for all clusters. The percent of cells expressing the gene (circle diameter) and the scaled average expression of the gene is indicated by the colour. **(C)** Cluster frequency analysis depicting the percentage of cells in each individual organoid that contributed to each cluster. **(A, D)** Individual hCOs (CO1-CO3) are plotted in the Uniform Manifold Approximation and Projection axis defined in panel (A). CP, choroid plexus; GLY, glycolytic signature; IPC, intermediate progenitor cells; oRG/astro, outer radial glial cells/astroglia; proRG, proliferative radial glial; RG, radial glial cells; RP, ribosomal protein.

**Figure S6.**
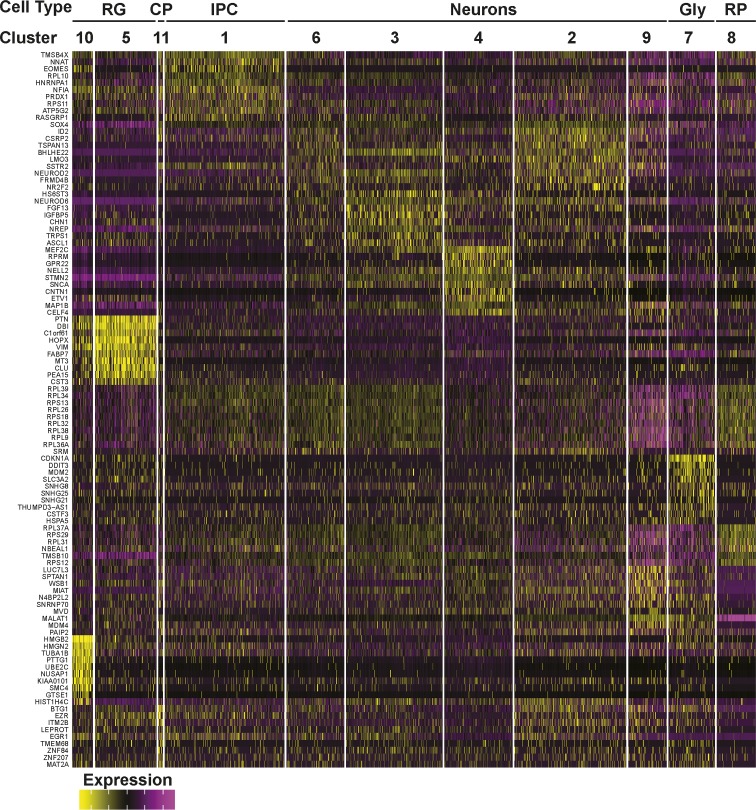
Gene expression heat map from 18-wk-old COs. The top 10 most differentially expressed genes per cluster are shown.

**Figure S7.**
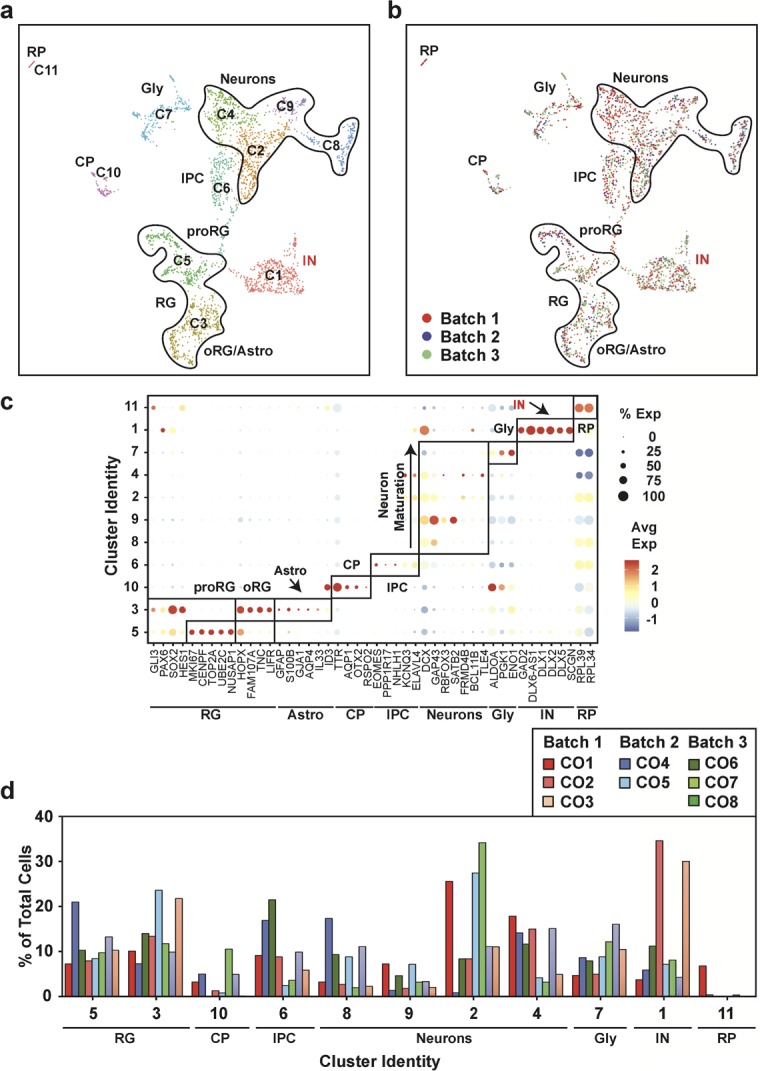
Uniform cell type composition in 24-wk-old human cerebral organoids is revealed by single-cell RNA sequencing. **(A)** Uniform Manifold Approximation and Projection plots from unsupervised clustering of single-cell RNA sequencing data from eight human cerebral organoids from three separate batches (CO1-CO8) (3,140 cells) are shown. Cluster identities are indicated. The emergence of an interneuron (IN; red text) and mature astrocyte population is observed. **(B)** A dot blot indicates the expression of cell type–specific marker genes for all clusters. The percent of cells expressing the gene (circle diameter) and the scaled average expression of the gene are indicated by the colour. **(C)** Cluster frequency analysis depicting the percentage of cells in each individual organoid that contributed to each cluster. **(A, D)** COs derived from each independent batch are plotted in the Uniform Manifold Approximation and Projection axis defined in panel (A). CP, choroid plexus; GLY, glycolytic signature; IN, interneurons; IPC, intermediate progenitor cells; oRG/astro, outer radial glial cells/astroglia; proRG, proliferative radial glial; RG, radial glial cells; RP, ribosomal protein.

**Figure S8.**
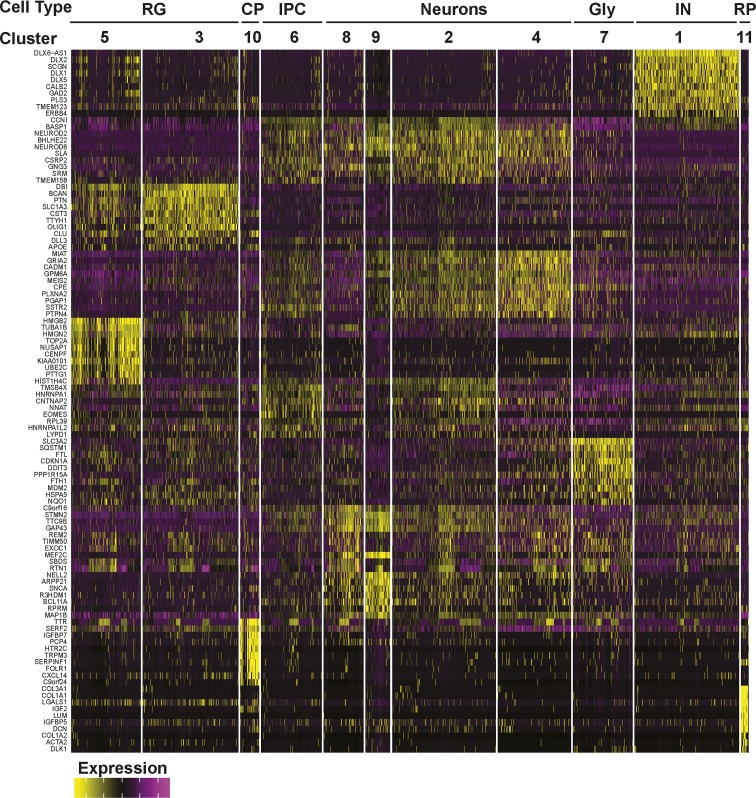
Gene expression heat map from 24-wk-old COs. The top 10 most differentially expressed genes per cluster are shown.

We next compared cell type composition in individual organoids to assess inter-organoid variability. For this, the percent of cells in each organoid that contributed to each of the 15 clusters was determined. This analysis revealed a remarkable conservation of cell type composition amongst the individual organoids (cluster frequency variation of <5%) even when derived from independent batches (Fig 3B and C). Importantly, all COs contained cells from all of the clusters, indicative of conserved developmental trajectories. Transcriptional profiling of older hCOs of 18 wk (three hCOs from three batches) and 24 wk (eight hCOs from three batches) was also performed (Fig S5–S8). Like 12-wk-old hCOs, cell type composition was similar between individual hCOs across multiple organoids and batches at both 18 and 24 wk, with a median cluster frequency variation of 3% and 15%, respectively (Figs S5A–D and S7A–D). Thus, even upon prolonged culturing, our optimized pipeline yields organoids of similar composition and morphology.

### Analysis of cell type diversity in maturing hCOs

**Figure 4.**
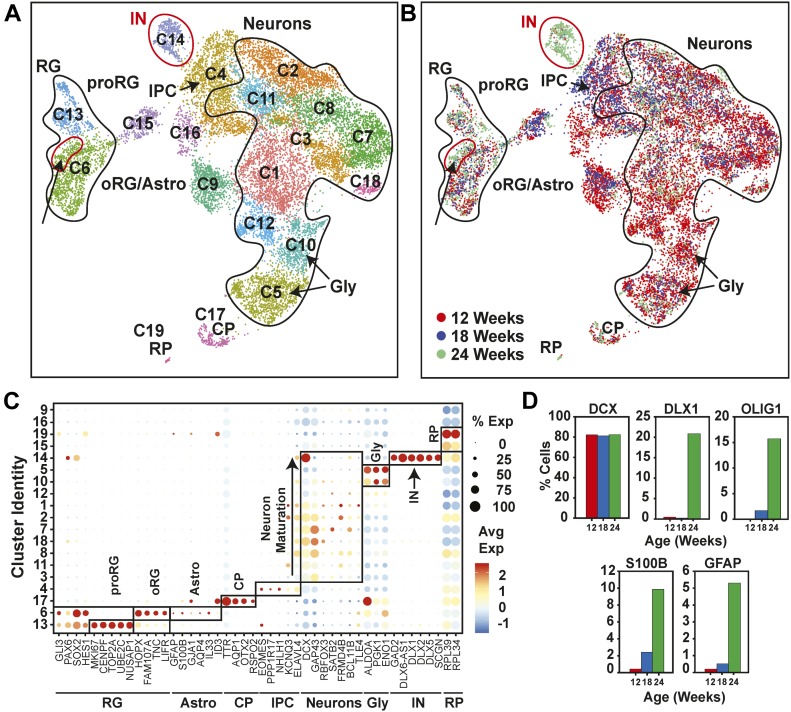
Characterization of cell type maturation in human cerebral organoids using single-cell RNA sequencing. **(A)** Unsupervised clustering of combined single-cell RNA sequencing data from 12-, 18-, and 24-wk-old COs was visualized in Uniform Manifold Approximation and Projection plots. Cell types appearing at 24 wk, including interneurons (IN) in cluster 14 (red circle) and oligodendrocyte progenitors in oRG/astroglial cluster 6, (arrow and red circle) are marked. Cluster identities are indicated. **(A, B)** Uniform Manifold Approximation and Projection present in (A) segregated by time point. Cells from 12 wk depicted in red, blue for 18 wk, and green for 24 wk. **(A, C)** A dot blot indicates the expression of cell type–specific marker genes for all clusters in panel (A). The percent of cells expressing the gene (circle diameter) and the scaled average expression of the gene is indicated by the colour. **(D)** The percent of cells with expression of the indicated cell lineage markers, including *DCX* (neurons), *S100B* (mature astrocytes), *GFAP* (astroglia/astrocytes), *DLX1* (interneurons), and *OLIG1* (oligodendrocyte precursors) and at 12, 18, and 24 wk time points is plotted. Note the general neuronal marker *DCX*, which is expressed similarly across the time points, was used as a reference. CP, choroid plexus; GLY, glycolytic signature; IN, interneurons; IPC, intermediate progenitor cells; oRG, outer radial glial cells; proRG, proliferative radial glial; RG, radial glial cells; RP, ribosomal protein.

**Figure S9.**
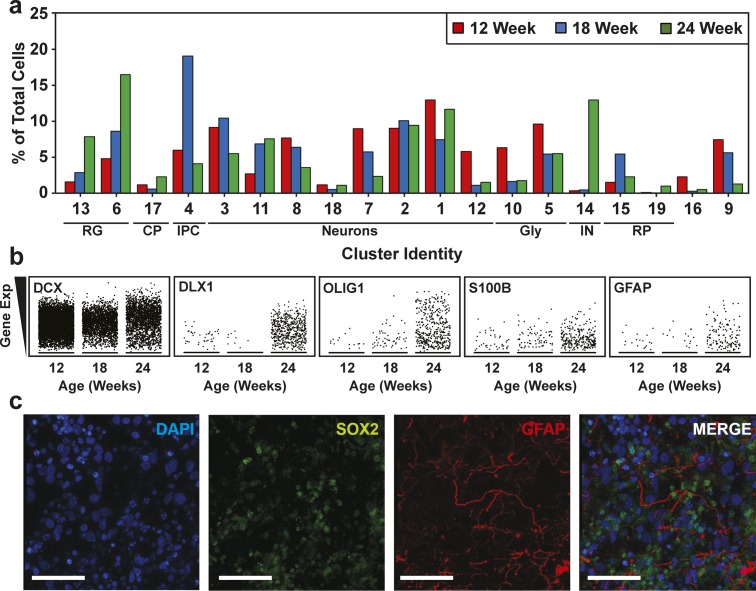
Cell type composition of human cerebral organoids (hCOs) changes with maturation. **(A)** Cluster frequency analysis depicting the percentage of cells at each time point that contributed to each cluster. **(B)** Scatterplots showing the expression of cell lineage markers, including DCX (neurons), S100B (mature astrocytes), GFAP (astroglia/astrocytes), DLX1 (interneurons), and OLIG1 (oligodendrocyte precursors) at 12, 18, and 24 wk time points. Note the general neuronal marker DCX, which is expressed similarly across the time points, was used as a reference. **(C)** Astrocyte in 24-wk-old hCOs. Localization of GFAP (radial glia and astrocytes) and SOX2 (radial glia) in hCOs at 24 wk of age, co-stained with DAPI, was visualized by immunofluorescence microscopy. Scale Bar = 50 μm. CP, choroid plexus; GLY, glycolytic signature; IN, interneurons; IPC, intermediate progenitor cells; oRG/astro, outer radial glial cells/astroglia; proRG, proliferative radial glial; RG, radial glial cells; RP, ribosomal protein.

**Figure S10.**
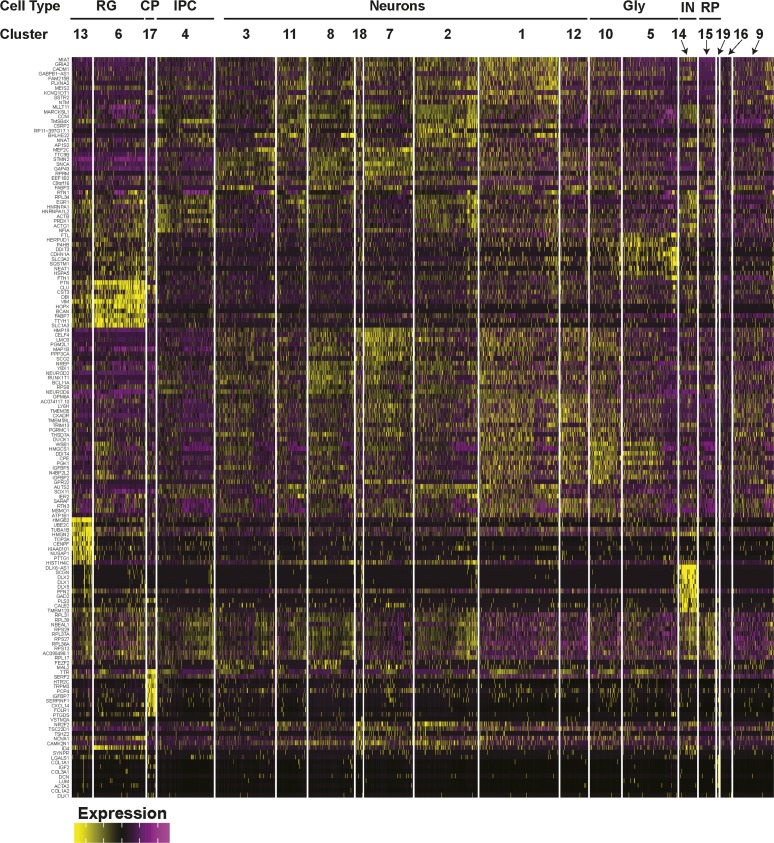
Gene expression heat map from combined 12-, 18-, and 24-wk-old COs. The top 10 most differentially expressed genes per cluster are shown.

To track cell composition in maturing organoids, the transcriptional profiles of 12-, 18-, and 24-wk-old organoids were combined and plotted in a single UMAP comprising 19 clusters (Figs 4A, S9A and B, and S10). Analysis of the relative contribution of cells from each time point (Figs 4B and C and S9A) revealed the emergence at 24 wk of a new cluster comprised interneurons (IN; expressing *DLX1*, *DLX2*, *DLX6*, *DLX6-AS*, *GAD1*, and *GAD2*). Of note, these interneurons lacked expression of *ISL1* and *EBF1*, suggesting caudal/medial identity, rather than lateral ganglionic eminence (Wamsley & Fishell, 2017). These INs cluster close to the intermediate progenitor cell population in the UMAP, suggesting they may have arisen from these progenitors (Fig 3D). We also noted another emerging subpopulation of cells within the RG/astroglia cluster (cluster 6; circle) in 24-wk-old hCOs (Fig 4A and B). This population was marked by *OLIG1* and *OLIG2* expression, suggesting an oligodendrocyte precursor population (Figs 4A–D and S9B). RG cells are known to produce both neurons and glia, although few cells (<1%) in the 12-wk-old hCOs expressed markers of mature astrocytes (*GFAP* and *S100B*; Figs 4D and S9B and C). However, cells with an astroglial identity (Astro) were detected within the oRG cluster starting at 18 wk (Fig S5, cluster 5) that further increased by 24 wk (Fig S7A, cluster 3), indicating the emergence of mature astroglia from RG cells (Figs 4D and S9B and C). Overall, this suggests that the rate of gliogenesis increases upon longer culturing, which parallels the temporal regulation of cortical development in vivo (Sauvageot & Stiles, 2002; Pinto & Gotz, 2007).

### Electrophysiological analysis

**Figure 5.**
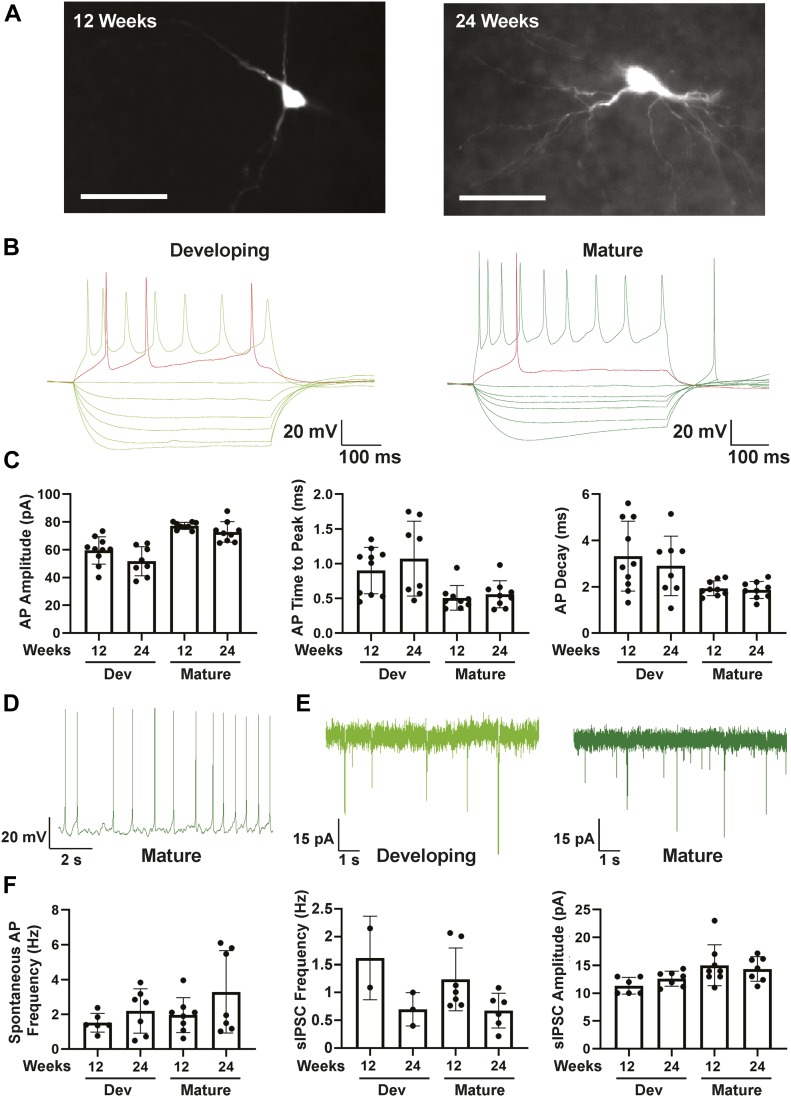
Electrophysiological analysis of 12- and 24-wk-old human cerebral organoids using whole cell patch clamping. **(A)** Images of recorded developing or mature neurons marked with biocytin taken using 40× water immersion objective are shown. Scale bar = 20 μm. **(B)** Representative traces from whole cell patch clamping of developing and mature neurons are shown. **(C)** Peak action potential amplitude ± SD, mean time to peak action potential amplitude ± SEM, and mean action potential decay time ± SEM, for individual recordings are plotted for 8–10 neurons per condition. **(D, E)** Characterization of spontaneous currents. **(D, E)** A representative trace of spontaneous AP firing in a mature neuron (D) and of mean frequency ± SEM of spontaneous excitatory postsynaptic currents in developing and mature neurons (E) are shown. **(F)** Frequency and amplitude of spontaneous IPSCs in developing and mature neurons are plotted as the mean ± SEM for 2–6 neurons per condition.

**Figure S11.**
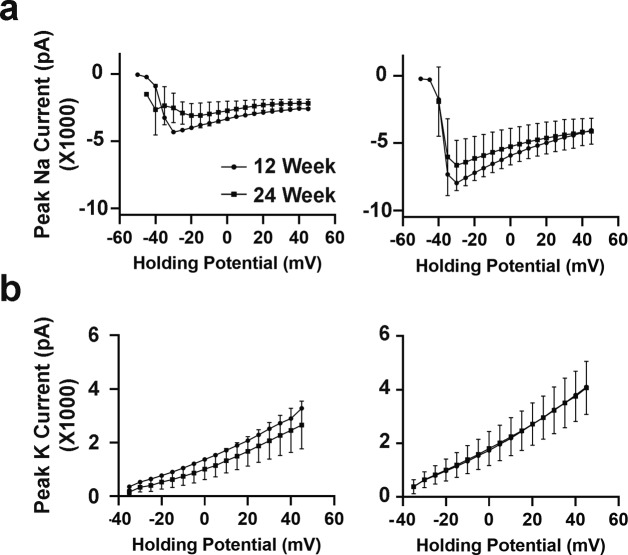
Electrophysiological analysis of 12- and 24-wk-old human cerebral organoids using whole cell patch clamping. **(A, B)** Na+ (A) and K+ (B) currents in developing and mature neurons analyzed in Fig 4 are plotted ± SEM.

To assess neuronal function, electrophysiological output was measured by whole cell patch clamping of individual neurons in fresh slices prepared from hCOs at 12 and 24 wk. Patched neurons displayed varying degrees of neurite networks as visualized by biocytin labelling (Fig 5A). Analysis of electrical properties identified three types of neurons, namely, immature, developing, and mature. Immature neurons did not fire action potentials (AP; data not shown), developing neurons, fired APs but with slow kinetics and small amplitudes, and mature neurons, fired APs with fast kinetics and high amplitudes and generated stable trains of spontaneous APs (Fig 5B and C). Both developing and mature neurons also displayed sodium and potassium currents, although they were smaller in the developing neurons (Fig S11). Moreover, only mature neurons generated stable spontaneous APs upon slight depolarization although both developing and mature neurons displayed similar spontaneous AP frequency and amplitude (Fig 5D–F). Of note, all three neuron classes were present in both 12- and 24-wk old hCOs, indicating that even at 12 wk, electrophysiologically mature neurons were present.

## Discussion

Human stem cell–derived cerebral organoids provide an unparalleled model system to study human neocortical development and associated disease processes. However, a key limitation for research applications is the considerable variability in shape/architecture and cell type composition present in individual organoids. This characteristic makes it particularly challenging to design experiments to address the effects of genetic variants, therapeutic candidates, and other perturbations on CO morphogenesis or function that allow statistically supported conclusions to be drawn. Here, we describe a protocol to efficiently generate human dorsal forebrain organoids with phenotypically uniform morphologies that are reproducibly comprised of similar proportions of cell types across independent batches. This highly reproducible model system is, thus, amenable for the study of pathways controlling early human brain development and disease processes.

Several protocols have been developed to generate cerebral organoids, including both directed and self-directed. For example, several groups use modified versions of a directed approach established by the Sasai group, sometimes referred to as cortical spheroids, in which small molecules that modulate the SMAD and Wnt pathways and the growth factors EGF and FGF2 are used to achieve directed differentiation (Kadoshima et al, 2013; Velasco et al, 2019; Yoon et al, 2019). Here, we selected a previously established method of self-patterned organoids (Lancaster et al, 2013; Lancaster & Knoblich, 2014a) as a starting point. Our work to standardize production revealed that optimization of the early steps of organoid generation, including the EB formation and neural induction phases, was key in ensuring the production of similar COs. This included normalizing EB morphogenesis using early removal of ROCKi and optimized FGF treatment, cell numbers, and well geometry and coatings. Finally, we found that efficient removal of Matrigel before spinning culture improved subsequent development. Applying this method, we demonstrated using single-cell profiling that our individual COs have extensive cell diversity but with similar proportions of cell types even across different batches. In contrast, the reported single-cell analysis of the originally established method revealed considerable variability in the proportions of cell types present in individual organoids, particularly across different batches (Quadrato et al, 2017). Furthermore, we observed uniform distribution of ventricular units, and that the cell type composition evident in 12-wk-old hCOs was maintained in older organoids, with additional cell types, including interneurons, mature astrocytes, and oligodendrocyte precursors emerging at 24 wk. Importantly, these new cell types were present in all organoids at similar proportions. Finally, our electrophysiological analysis revealed the presence of mature, electrically active neurons.

Our optimized self-directed protocol that generates highly reproducible hCOs complements those reported in other recent studies that use a directed patterning approach involving small molecule inhibitors and growth factors (Kadoshima et al, 2013; Velasco et al, 2019; Yoon et al, 2019). The availability of several robust platforms to produce highly similar hCOs, including the one described herein, thus allows for systematic molecular and cellular characterization of the development of the human cortex and how disease-causing mutations alter development and perhaps also homeostatic events.

## Materials and Methods

### Cell line generation and culturing

H9 and H1 ESCs were cultured on plates coated with hESC-grade Matrigel (#CA89050-192; VWR) at 37°C and 5% CO_2_ in mitotically arrested MEF-conditioned media, DMEM/F12 (#11330057; Life Technologies), 20% KnockOut Serum Replacement (KSR, #A3181502-02; Life Technologies), 2% MEM-NEAA, 55 μM β-mercaptoethanol (#21985-023; Life Technologies), and 4 ng/ml basic FGF (bFGF, #100-18B; Peprotech). The medium was changed daily and the cells were split at a 1:6 ratio using Collagenase IV (#07909; Stemcell Technologies) every 5–6 d.

### Generation of cerebral organoids

ESCs were singularized using TrypLe Select (#12563011; Life Technologies) and resuspended at 80,000 cells/ml in EB media (DMEM/F12, 20% KSR, 2% MEM-NEAA, 55 μM β-mercaptoethanol) with 4 ng/ml bFGF and 50 μM Y-27632 (Y27) (#S1049; Selleck Chem). The cells (12,000 cells/well) were plated in 96-well V-bottom nonbinding plates (#651970; Greiner Bio-One), and on Day 2, fresh EB medium containing 2 ng/ml bFGF was added. On Day 5, healthy EBs with a diameter of 425–475 μm were transferred to 24-well ultralow attachment plates (#CLS3473; Corning) in 500 μl neural induction medium (DMEM/F12, 1% N2, 1% MEM-NEAA, 1% GlutaMax, and 1 μg/ml heparin [#H3393; Sigma-Aldrich]), and 48 h later, an additional 500 μl of neural induction medium was added. EBs were transferred to pre-warmed four-well tissue culture plates, excess medium was aspirated, and freshly thawed growth factor reduced Matrigel (30 μl) was added on top of EBs. Plates were transferred to a 37°C CO_2_ incubator for 10 min to allow the Matrigel to polymerize, and then 500 μl of cerebral organoid differentiation media without vitamin A (CDM − Vit A; 48% DMEM/F12, 48% neurobasal [#21103049; Life Technologies], 0.5% MEM-NEAA, 1% GlutaMax, 0.5% N2, 1% B27 without vitamin A [#12587001; Life Technologies], 2.5 μM insulin, and 192.5 μM β-mercaptoethanol) was added to each well. On Day 11, the medium was replaced, and on Day 13, spheroids containing ring-like structures were extracted using a Scalpel (#1000044; Thermo Fisher Scientific), ensuring that excess Matrigel is removed. Organoids (maximum of three per well) were transferred to a six-well tissue culture plate containing 3 ml of CDM + Vit A (CDM with 1% B27 containing Vit A) and then placed on an orbital shaker (#88881101; Thermo Fisher Scientific) at 90 rpm (9.5 mm radius) in a CO_2_ incubator for the duration of culturing. The medium (2 ml) was replaced every 72 h for the first 30 d and then increased to 3 ml for the duration of culture. The plates were replaced every 30 d to prevent buildup of debris. Spheroid formation efficiency was tested using nonbinding V-bottom plates (#651970; Greiner Bio-One), Aggrewell800 plates (#38421; StemCell Tech) coated with Anti-Adherence Rinsing Solution (#07010; StemCell Tech), U-bottom non-treated polystyrene plates (#168136; Thermo Fisher Scientific), and U-bottom Ultra-Low Attachment plates (#4515; Corning).

### Immunofluorescence microscopy

COs were washed three times for 5 min in 5 ml of PBS in 5-ml round-bottom tubes (#1152367; BD Falcon) and then fixed overnight in 4% PFA at 4°C on a rocker. The samples were washed three times for 5 min in 0.1% Tween20 in PBS (PBST) and then sequentially soaked in 15% sucrose solution for 2 h and then 30% sucrose solution overnight (4°C). Organoids were transferred to a cryomold containing OCT (#95057-838; VWR) and flash-frozen in liquid nitrogen using a stainless steel bucket containing 2-methylbutane (#M32631; Sigma-Aldrich). The samples were sectioned to 20 μm using a Leica CM3050S cryostat, mounted on SuperFrost Plus Microscope Slides (#22-037-246; Thermo Fisher Scientific), and heated for 15 min at 45°C. The samples were washed three times in PBST at room temperature, blocked, and permeabilized for 1 h at room temperature in 2% BSA and 0.5% Triton-X100 diluted in PBST (PBSTx) in a humidified chamber. Primary antibodies listed in Table S2 were diluted in cold PBST containing 0.5% BSA and incubated overnight at 4°C, washed three times for 5 min in PBST at room temperature, and then incubated in Invitrogen DyLight Secondary Antibodies (1:500) and DAPI (1:2,000) in PBST containing 0.5% BSA overnight at 4°C. The slides were washed in PBST three times for 10 min and mounted using MOWIOL-DABCO Mounting Media (#10891; Sigma-Aldrich) and then dried at room temperature overnight in a dark box. The samples were imaged using a Nikon T2i microscope with a Hamamatsu confocal camera or Zeiss CSU-X1 confocal microscope. Images were processed using NIS elements (Nikon Elements) software or Volocity (PerkinElmer) software, respectively.

Table S2 Antibody list.

### Paraffin embedding

COs were washed two times for 5 min at room temperature in 5 ml of PBS in 5-ml round-bottom tubes (#1152367; BD Falcon) and then fixed overnight in 4% PFA at 4°C on a rocker. The samples were washed three times for 15 min in PBS and then sequentially dehydrated in 40%, 70%, and 95% ethanol diluted in ddH_2_O for 1 h each, 95% ethanol overnight, and then three times for 1 h in 100% ethanol at 4°C on a rocker. COs were then soaked in xylene for 5–10 min at room temperature in biopsy cassettes (#M506-3; Simport). The samples were air-dried for 2–3 min and washed twice in melted paraffin (#39602004; Leica) at 60°C for 1 h and once overnight. The samples were embedded into paraffin blocks within 16 h, sectioned using 80-mm microtome blades (MB35; Thermo Fisher Scientific) at 14 or 20 μm on a semiautomated rotary microtome (RM2245; Leica), mounted on SuperFrost Plus Microscope Slides in distilled H_2_O heated to 45°C, and then dried overnight at 45°C and stored at 4°C.

### Hematoxylin and eosin staining

Paraffin slides were deparaffinized by two washes of xylene, 100%, 100%, 95%, and 70% ethanol for 5 min each. Deparaffinized or cryopreserved slides were soaked for 5 min in dH_2_O, stained with Harris’ Hematoxylin (#HHS32; Sigma-Aldrich) for 3 min, rinsed in warm running tap water for 4 min, and then de-stained in 0.3% acid ethanol (70% ethanol and 0.3% HCl) for 30 s and rinsed in warm running tap water for 5 min. The samples were dipped in the bluing agent 0.1% sodium carbonate (#1613757; Sigma-Aldrich), washed in running tap water for 2 min, and then counterstained in a working solution of 0.25% eosin (in 80% ethanol and 0.5% glacial acetic acid) for 20 s. The slides were sequentially dehydrated for 1 min in 70%, 95%, and 100% ethanol and for 5 min in xylene or Histo-Clear II (#HS-202; National Diagnostics), mounted using Histomount (#008030; Thermo Fisher Scientific), and left to dry overnight before imaging. H&E images were taken on a dissection scope with a mounted camera or with a 20× objective on Axio Scan.Z1 (Zeiss) and processed using Zen (Zeiss) software.

### Sample preparation for scRNA-seq

COs were washed three times for 1 min each in with 5 ml of room-temperature PBS, cut into fragments, digested with Accutase at 37°C for 20 min, with pipetting every 10 min to facilitate cell dissociation. The cell suspension was passed through a 40-micron filter, which was then washed once with 2 ml of fresh Accutase to collect remaining single cells and then centrifuged at 120 rcf. The pellet was resuspended in 5 ml of Accutase at room temperature. Live cells were counted using trypan blue staining, centrifuged at 120 rcf for 5 min, and single cells were resuspended in CDM + Vit A at 500,000–1,000,000 cells/ml and trypan blue–positive cells recounted. The samples were placed on ice and then subjected to barcoding using the 10× Chromium platform following the manufacturer’s recommendations. cDNA libraries were sequenced on either Illumina’s HiSeq 3000 or a NovaSeq 6000.

### Analysis of scRNA-seq data

scRNA-seq were processed with Cell Ranger (V2.1) software (10× Genomics) to perform demultiplexing, unique molecular identifier collapsing, demultiplexing, and alignment to the GRCh38 human transcriptome. Post-processing of the raw Cell Ranger output matrix was performed using an in-house pipeline (Ayyaz et al, 2019). Data processing including cell filtering using a stringent filter, gene filtering, and data normalization for individual samples were performed with scater (McCarthy et al, 2017). Merging of multiple samples into a combined object, unsupervised clustering, visualizations, and differential expression analysis was performed using Seurat (Butler et al, 2018; Stuart et al, 2019). In Seurat, UMAP was used as the preferred dimensionality reduction method (Becht et al, 2018) whereas MAST was used for differential expression analysis (Finak et al, 2015). Batch correction between multiple batches in the same time point as well as between time points was performed using Harmony (Korsunsky et al, 2018 *Preprint*). All data plotting and analyses were performed in R (www.R-project.org).

### Electrophysiological recordings

COs (500 μm) were embedded in 0.4% low-melting agarose (#16520100; Thermo Fisher Scientific) diluted in PBS and then sectioned using a Leica VT1200S vibratome. The slices were perfused at a constant rate of 2 ml/min with oxygenated, warmed recording artificial cerebrospinal fluid solution, containing 124 mM NaCl, 25 mM NaHCO_3_, 2.5 mM KCl, 2.5 mM MgCl_2_, 1.2 mM CaCl_2_, and 10 mM glucose at a temperature 32°C ± 1°C. Visually guided whole-cell patch-clamp recordings were obtained from neurons with a patch solution containing 120 mM K-gluconate, 20 mM KCl, 10 mM Hepes, 2 mM MgCl_2_, 2 mM Mg_2_ATP, 0.3 mM NaGTP, 7 mM phosphocreatine, and 0.6 mM EGTA (pH = 7.2, 295 mOsm). Biocytin was routinely added to patch solution to reveal the morphology of recorded neurons. Immediately after recordings, the slices were fixed in 4% PFA, and immunostaining for biocytin was performed using streptavidin–Alexa 546–conjugated antibodies as described previously (Khlghatyan et al, 2019).

## Data Availability

The accession number for the gene expression data reported in this article is GEO: GSE137877.

## Supplementary Material

Reviewer comments
