# Supplementary material for: Robust production of uniform human cerebral organoids from pluripotent stem cells
Source: Life Sci Alliance. 2020 Apr 17;3(5):e202000707. doi: 10.26508/lsa.202000707 (PMC7167289; doi:10.26508/lsa.202000707)
Supplement: Reviewer comments [file LSA-2020-00707_review_history.pdf]

Robust Production of Uniform Human Cerebral Organoids from Pluripotent Stem Cells

Adam Sivitilli, Jessica Gosio, Bibaswan Ghoshal, Alesya Evstratova, Daniel Trcka, Parisa Ghiasi, Javier Hernandez, Jean-Martin Beaulieu, Jeffrey Wrana and Liliana Attisano

DOI: 10.26508/lsa.202000707

Corresponding author(s): Liliana Attisano (University of Toronto)

Review timeline:

Submission Date:	2020-03-18
Editorial Decision:	2020-03-18
Revision Received:	2020-04-03
Editorial Decision:	2020-04-06

Scientific Editor: Andrea Leibfried

Transaction Report:

(Note: With the exception of the correction of typographical or spelling errors that could be a source of ambiguity, letters and reports are not edited. The original formatting of letters and referee reports may not be reflected in this compilation.)

No full Peer Review Process File is available with this article, as the authors have chosen not to make the review process public in this case.

Please note that the manuscript was previously reviewed at another journal and the reports were taken into account in the decision-making process at Life Science Alliance.

1st Editorial Decision 18 March 2020

RE: Life Science Alliance Manuscript #LSA-2020-00707-T

Liliana Attisano
Toronto, University of
Dept. of Biochemistry
Donnelly Centre, Room 1008
160 College Street
Toronto, Ontairo M5S 3E1
Canada

Dear Dr. Attisano,

Thank you for submitting your revised manuscript entitled "A Human Cerebral Organoid Allograft Model for Glioblastoma". Your manuscript was reviewed at another journal before and you revised it in response to the feedback received and you outlined how you could further address some concerns. The reviewer reports and your point-by-point response were transferred to us by the editors with your permission.

We appreciate that you concentrated in the revised version of your manuscript on the mature methods part, which was deemed robust by the reviewers. We think it would be great to include the bFGF dose response analyses at various cell seeding densities. We would thus like to invite you to submit a final version of your manuscript to us for publication here. In addition to the experiment you offered to include in response to reviewer #4's concern, we would like to ask you to include the other amendments you propose to address the issues noted by this reviewer. In addition, please pay attention to the following:

- Please make sure that the authors listed in our submission system and those listed in the manuscript file are identical
- Please link your ORCID iD to your profile in our submission system, you should have received an email with instructions on how to do so
- Please provide a summary blurb for your method and individual author contributions in our submission system when submitting the final files
- Please provide your manuscript file in docx format
- Please make sure that others can easily use the method, I would like to encourage you to check the methods section again to ensure that re-use by others is easy
- Please upload all figures, including supplementary figures, as individual files and without legends; all legends should be in the main manuscript docx file; the two supplementary tables can get uploaded as docx files
- Please either callout all sub-panels of a figure or none: Fig S5c is currenty called out but not S5a, b, d; figures S7c is currently called out but not S7a, b, d
- Please note that the legends for Fig S2 and S3 do not match the figures (in terms of panels mentioned)
- Please add a scale bar to Fig S2a
- It was not clear to me which supplementary files are the most recent ones - note that there is currently not a callout in the manuscript text to Fig S12 and S13

If you are planning a press release on your work, please inform us immediately to allow informing our production team and scheduling a release date.

To upload the final version of your manuscript, please log in to your account:

https://lsa.msubmit.net/cgi-bin/main.plex
You will be guided to complete the submission of your revised manuscript and to fill in all necessary information. Please get in touch in case you do not know or remember your login name.

To avoid unnecessary delays in the acceptance and publication of your paper, please read the following information carefully.

A. FINAL FILES:

These items are required for acceptance.

-- An editable version of the final text (.DOC or .DOCX) is needed for copyediting (no PDFs).

-- High-resolution figure, supplementary figure and video files uploaded as individual files: See our detailed guidelines for preparing your production-ready images, http://www.life-science-alliance.org/authors

-- Summary blurb (enter in submission system): A short text summarizing in a single sentence the study (max. 200 characters including spaces). This text is used in conjunction with the titles of papers, hence should be informative and complementary to the title. It should describe the context and significance of the findings for a general readership; it should be written in the present tense and refer to the work in the third person. Author names should not be mentioned.

B. MANUSCRIPT ORGANIZATION AND FORMATTING:

Full guidelines are available on our Instructions for Authors page, http://www.life-science-alliance.org/authors

We encourage our authors to provide original source data, particularly uncropped/-processed electrophoretic blots and spreadsheets for the main figures of the manuscript. If you would like to add source data, we would welcome one PDF/Excel-file per figure for this information. These files will be linked online as supplementary "Source Data" files.

Submission of a paper that does not conform to Life Science Alliance guidelines will delay the acceptance of your manuscript.

It is Life Science Alliance policy that if requested, original data images must be made available to the editors. Failure to provide original images upon request will result in unavoidable delays in publication. Please ensure that you have access to all original data images prior to final submission.

The license to publish form must be signed before your manuscript can be sent to production. A link to the electronic license to publish form will be sent to the corresponding author only. Please take a moment to check your funder requirements.

Reviews, decision letters, and point-by-point responses associated with peer-review at Life Science Alliance will be published online, alongside the manuscript. If you do want to opt out of having the reviewer reports and your point-by-point responses displayed, please let us know immediately.

Thank you for this interesting contribution, we look forward to publishing your paper in Life Science Alliance.

Sincerely,

Andrea Leibfried, PhD
Executive Editor
Life Science Alliance
Meyerhofstr. 1
69117 Heidelberg, Germany
t +49 6221 8891 502
e a.leibfried@life-science-alliance.org
www.life-science-alliance.org

2nd Editorial Decision 6 April 2020

RE: Life Science Alliance Manuscript #LSA-2020-00707-TR

Liliana Attisano
Toronto, University of
Dept. of Biochemistry
Donnelly Centre, Room 1008
160 College Street
Toronto, Ontairo M5S 3E1
Canada

Dear Dr. Attisano,

Thank you for submitting your Methods entitled "Robust Production of Uniform Human Cerebral Organoids from Pluripotent Stem Cells". It is a pleasure to let you know that your manuscript is now accepted for publication in Life Science Alliance. Congratulations on this interesting work.

The final published version of your manuscript will be deposited by us to PubMed Central upon online publication.

Your manuscript will now progress through copyediting and proofing. It is journal policy that authors provide original data upon request.

Reviews, decision letters, and point-by-point responses associated with peer-review at Life Science Alliance will be published online, alongside the manuscript. If you do want to opt out of having the reviewer reports and your point-by-point responses displayed, please let us know immediately.

IMPORTANT: If you will be unreachable at any time, please provide us with the email address of an alternate author. Failure to respond to routine queries may lead to unavoidable delays in publication.

Scheduling details will be available from our production department. You will receive proofs shortly before the publication date. Only essential corrections can be made at the proof stage so if there are any minor final changes you wish to make to the manuscript, please let the journal office know now.

DISTRIBUTION OF MATERIALS:
Authors are required to distribute freely any materials used in experiments published in Life Science Alliance. Authors are encouraged to deposit materials used in their studies to the appropriate repositories for distribution to researchers.

You can contact the journal office with any questions, contact@life-science-alliance.org

Again, congratulations on a very nice paper. I hope you found the review process to be constructive and are pleased with how the manuscript was handled editorially. We look forward to future exciting submissions from your lab.

Sincerely,

Andrea Leibfried, PhD

Executive Editor
Life Science Alliance
Meyerhofstr. 1
69117 Heidelberg, Germany
t +49 6221 8891 502
e a.leibfried@life-science-alliance.org
www.life-science-alliance.org
